# Novel polymorphism of interleukin-18 associated with greater inflammation after cardiac surgery

**DOI:** 10.1186/cc7698

**Published:** 2009-01-29

**Authors:** David M Shaw, Ainsley M Sutherland, James A Russell, Samuel V Lichtenstein, Keith R Walley

**Affiliations:** 1 Critical Care Research Laboratories, Heart + Lung Institute, University of British Columbia, 1081 Burrard Street, Vancouver, BC, V6Z 1Y6, Canada

## Abstract

**Introduction:**

Interleukin (IL)-18 is a key modulator of the cytokine response that leads to organ dysfunction and prolonged intensive care unit (ICU) stay after cardiopulmonary bypass surgery. We hypothesised that variation in the pro-inflammatory gene IL-18 is associated with adverse clinical outcome because of a more intense inflammatory response.

**Methods:**

Haplotypes of the IL-18 gene were inferred from genotypes of 23 Coriell Registry subjects. Four haplotype tag single nucleotide polymorphisms (-607 C/A, -137 G/C, 8148 C/T and 9545 T/G) identified four major haplotype clades. These polymorphisms were genotyped in 658 Caucasian patients undergoing cardiopulmonary bypass surgery. Clinical phenotypes were collected by retrospective chart review.

**Results:**

Patients homozygous for the T allele of the 9545 T/G polymorphism had an increased occurrence of prolonged ICU stay (6.8% for TT genotype versus 2.7% for GG or GT genotype; p = 0.015). Patients homozygous for the T allele also had increased occurrence of low systemic vascular resistance index (62%) compared with the GG and GT genotypes (53%; p = 0.045). Patients homozygous for the T allele had increased serum IL-18 concentrations 24 hours post-surgery (p = 0.018), increased pro-inflammatory tumour necrosis factor alpha concentrations (p = 0.014) and decreased anti-inflammatory serum IL-10 concentrations (p = 0.018) 24 hours post-surgery.

**Conclusions:**

The TT genotype of the IL-18 9545 T/G polymorphism is associated with an increased occurrence of prolonged ICU stay post-surgery and greater post-surgical inflammation. These results may be explained by greater serum IL-18, leading to greater pro-versus anti-inflammatory cytokine expression.

## Introduction

The balance of pro-inflammatory (e.g. tumour necrosis factor alpha (TNF-α)) and anti-inflammatory (e.g. interleukin (IL)-10) cytokine gene expression is highly correlated with organ dysfunction and adverse outcome after cardiopulmonary bypass (CPB) surgery [1]. IL-18 is a key cytokine regulator of this balance that, among other clinical and cytokine measures, stands out as predictive of organ dysfunction and adverse outcomes after CPB [2]. IL-18 acts with IL-12 in a synergistic fashion to stimulate the release of interferon-gamma (IFN-γ) from lymphocytes [3]. High serum levels of IL-18 are associated with increased production of the pro-inflammatory cytokine TNF-α [4] and decreased production of the anti-inflammatory cytokine IL-10 [5]. Serum levels of IL-18 increase in response to CPB surgery [2] and in other inflammatory conditions such as sepsis [6] and type 1 diabetes [7].

Inflammatory gene polymorphisms have been linked to the intensity of the post-operative inflammatory response and to clinical outcomes after CPB surgery [8]. Therefore, we postulated that IL-18 gene single nucleotide polymorphisms (SNPs) may be important in recovery from CPB surgery. Two polymorphic loci within the IL-18 gene have been investigated for associations between genotype and serum concentrations of IL-18 (intermediate phenotype) as well as associations between genotype and clinical outcome (phenotype). The -607 C/A and -137 G/C SNPs were initially discovered to influence promoter activity of the IL-18 gene [9]. The -137 C allele has been associated with adverse outcomes and higher serum IL-18 cytokine levels, and the -607 A allele has been associated with improved outcomes and lower serum IL-18 levels in type 1 diabetes [3].

Our study design was based on the above observation that increased serum IL-18 is associated with adverse outcomes after CPB, including cardiovascular dysfunction [2], associated with increased serum TNF-α and decreased serum IL-10 [10]. Therefore, we tested the hypothesis that IL-18 gene polymorphisms are associated with adverse outcome, as measured by prolonged stays in the intensive care unit (ICU) (primary clinical outcome) and cardiovascular dysfunction (secondary clinical outcome). We then confirmed that the polymorphism is associated with increased serum concentrations of IL-18 and altered serum concentrations of TNF-α and IL-10 post-CPB, thus providing a possible explanation for our clinical results.

## Materials and methods

### Patient cohort

All patients admitted to the cardiac surgery ICU of St Paul's Hospital in Vancouver having elective CPB surgery between February 2001 and December 2003 were eligible for entry into this study. For inclusion, 890 were screened, of which 815 had CPB pump-driven circulation of blood during first-time elective coronary artery bypass surgery. Of these, we restricted our analyses to the 658 patients who were Caucasian in order to decrease the potentially confounding influence of population admixture secondary to ethnic diversity on associations between genotype and phenotype. All 658 patients were successfully genotyped for four polymorphisms in the IL-18 gene and made up our final cohort for analysis.

Our Research Ethics Board approved analysis of fully anonymous data and, for DNA samples, collection of blood that was being discarded as part of routine clinical care in a consecutive cohort of all patients meeting inclusion criteria and admitted to our cardiac surgical ICU. Using this fully anonymous study design the Research Ethics Board of Providence Health Care and the University of British Columbia approved this study and waived informed consent.

### Primary clinical phenotype

This cohort was confined to patients undergoing first-time elective coronary artery bypass surgery, so a mortality rate endpoint would be ineffective unless sample sizes were very large. Many authors, therefore, use prolonged ICU stay as a measure of adverse outcome [11,12]. Most recently, Nakasuji and colleagues found that prolonged ICU stay of more than three days was a sensitive and specific measure of adverse outcome that reflected measures of cardiovascular and pulmonary organ failure [11]. Lawrence and colleagues found that the Parsonnet score with a maximum predictive efficiency was a score of 10. Patients having a Parsonnet score of 0 to 9 had a mean ICU stay of about 1.5 days, while those having a score of 10 or more had a mean ICU stay of about three days [12]. Therefore, we used the proportion of patient having a post-operative ICU stay of greater than or equal to three days (72 hours) as our primary outcome variable. In our institution, the decision to discharge patients from the ICU after CPB surgery is protocol driven and genotype was unknown to care providers, making this measurement an unbiased outcome measurement for this study, particularly because genotype was fully blinded and not measured until after complete clinical data had been collected.

### Secondary clinical phenotype

Kristof and Magder [13] identified low post-CPB systemic vascular resistance index (SVRI) as a clinical manifestation of systemic inflammation. This vasodilatory syndrome is associated with related parameters such as longer cross-clamp times and lower post-CPB platelet count [13]. We used Kristof and Magder's definition of two consecutive SVRI measurements less than 1800 dyne.s/cm^5^/m^2 ^(SVRI = ((MAP-CVP) × 80)/CI, where MAP is the mean arterial pressure, CVP is the central venous pressure and CI is the cardiac index) as a secondary clinical phenotype.

### Intermediate phenotype

Serum concentrations of cytokines are useful intermediate phenotypes to test for biologically plausible explanations for genotype – clinical phenotype associations. We measured serum IL-18, TNF-α and IL-10 concentrations in an overlapping subset of patients (n = 44) within the current cohort for whom serum was available from a related observational study. Inclusion and exclusion criteria for this cohort were identical. In our own preliminary time course experiments we found that serum IL-18 concentrations peaked at 24 hours post-CPB. Therefore, we measured serum IL-18, TNF-α and IL-10 at this post-operative time point. Serum IL-18 and TNF-α were measured by ELISA (R&D Systems, Minneapolis, MN for IL-18; BD PharMingen, San Diego, CA for TNF-α). Serum IL-10 was measured using the Luminex bead-based bioassay system (Luminex Corp, Austin, TX).

### Tag single nucleotide polymorphism selection

**Figure 1:**
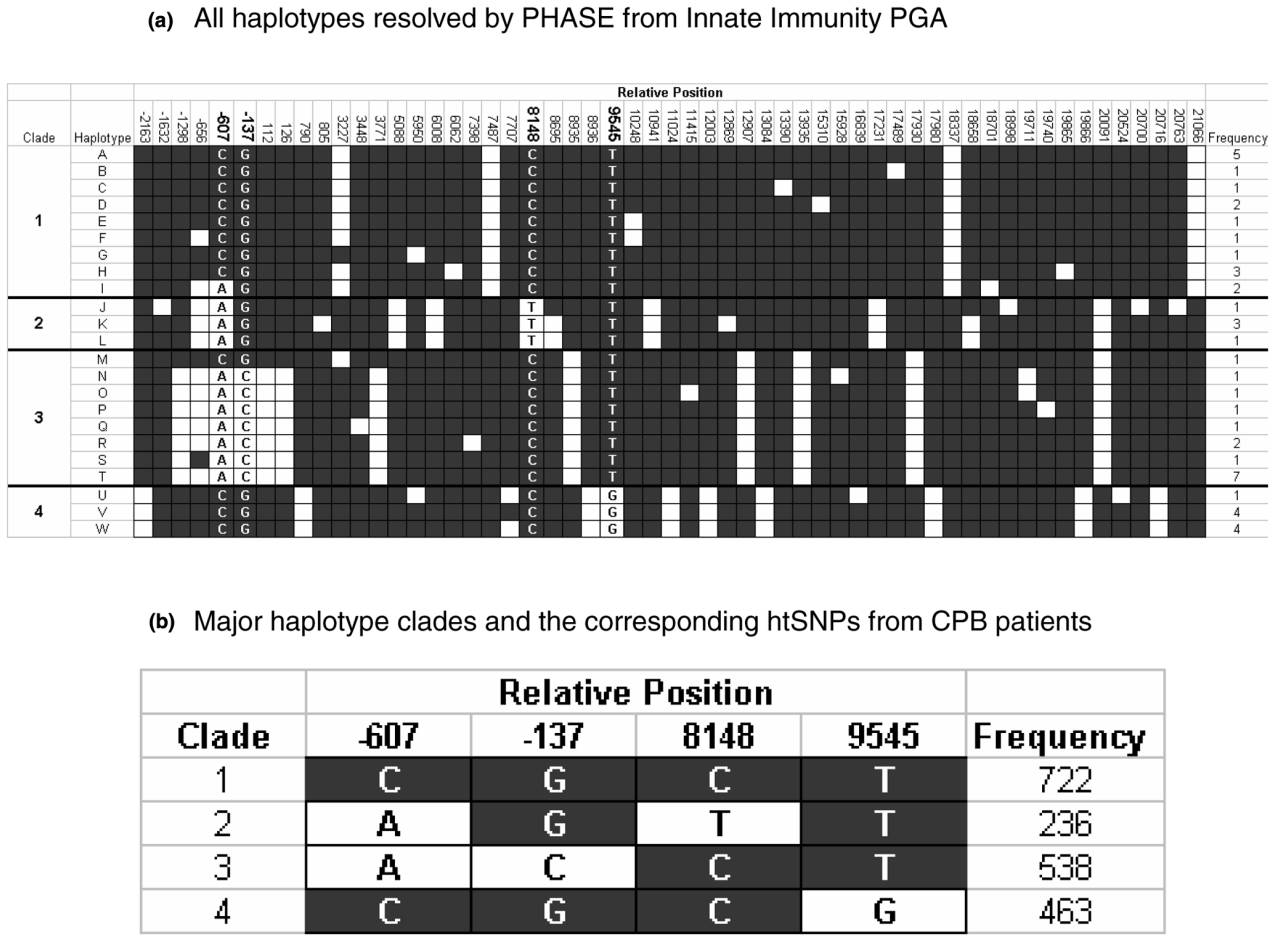
**Haplotypes of the IL-18 gene**. **a) Haplotype diagram of the interleukin (IL)-18 gene**. Haplotypes were inferred from unphased genotype data of 23 unrelated healthy Caucasians by the PHASE v2.0 program. Each column represents a polymorphic locus in the IL-18 gene, and is coded as either the common allele (black square) or the rare allele (white square). The position of each polymorphic locus relative to the start site of translation is indicated at the top of the diagram. Each row indicates a unique haplotype inferred from genotype data. The clustering of similar haplotypes into clades is done as per the phylogenetic relation among haplotypes given by the MEGA2 software package. The positions chosen for genotyping (-607, -137, 8148 and 9545) are indicated in the figure by bold numbering of the relative position and letter designations for the alleles within the diagram. **b) Haplotype diagram of the interleukin-18 gene in the patient population under study**. Haplotypes were inferred from unphased genotype data of 658 unrelated Caucasians making up the study cohort using the PHASE v2.0 program. Allele and clade designations within the diagram are as in Figure 1a. Haplotype frequency within the patient cohort is indicated to the right of each haplotype. Only haplotype clades with frequency greater than 5% are represented.

To determine IL-18 gene haplotypes we used unphased genotype data from the University of Arizona's Innate Immunity Program in Genomics Application website. We used PHASE v 2.0 [14] to infer haplotype from unphased genotype data. The resulting haplotypes were clustered into four groups of similar haplotypes (clades) using the molecular evolutionary genetic analysis software package MEGA2 [15]. The program LDSelect [16] was used to select a set of maximally informative haplotype tag (ht) SNPs with the restriction that the literature SNPs -607 C/A and -137 G/C be included. Four htSNPs were chosen to differentiate the four haplotype clades: -607 C/A (rs1946518), -137 G/C (rs187238), 8148 C/T (rs360722) and 9545 T/G (rs5744249) (Figure 1).

### Genotyping

DNA was extracted from peripheral blood samples using a QIAamp DNA Blood Maxi Kit (Qiagen Inc. Canada, Mississauga, ON, Canada). SNP genotypes were determined using the 5' nuclease, or Taqman PCR method (Applied Biosystems; Foster City, CA, USA) [17]. These htSNPs were then genotyped in the 658 patients of our cohort undergoing CPB.

### Statistics

Differences in continuous variables were assessed using Student's *t*-test for two groups or an analysis of variance for more than two groups for normally distributed data, and a Mann-Whitney U Test or Kruskal-Wallis H Test for non-normally distributed data. Fisher's exact test was used to test for significant differences in discrete variables. Data are reported as mean ± standard error (SE). A p ≤ 0.05 was taken to indicate a significant difference. Analysis was performed using SPSS v11.5 (SPSS, Chicago, IL, USA). Allele frequencies were tested for Hardy-Weinberg equilibrium using the test of Guo and Thompson [18] and were all found to be in Hardy-Weinberg equilibrium.

Work by Long and Langley [19] indicates that on the order of 500 individuals are sufficient to detect the presence of causative polymorphisms having small effect on outcomes. Thus, we have included 658 individuals in our study population.

## Results

### Selection of the 9545 T/G SNP

**Figure 2:**
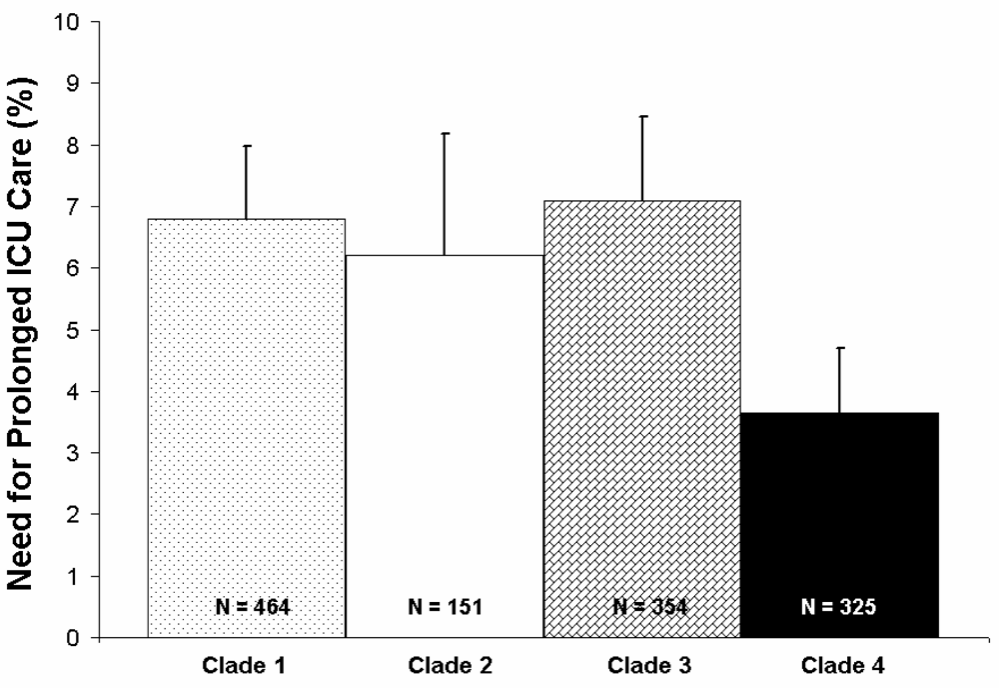
**Prolonged intensive care unit (ICU) stay after cardiopulmonary bypass (CPB) by interleukin (IL)-18 9545 T/G genotype in Caucasian patients who underwent on-pump CPB surgery**. Percentage of patients having prolonged ICU stay (greater than 72 hours) are indicated on the Y-axis and IL-18 genotype on the X-axis. Patients homozygous for the T allele of the IL-18 9545 T/G SNP had increased occurrence of prolonged ICU stay, compared with patients having a TG or GG genoytpe.

Using PHASE, we found four main haplotypes occurring at a frequency greater than 5% in our patient cohort based on the four chosen htSNPs (Figure 1b). As an initial screen, we examined the occurrence of our primary clinical outcome (prolonged ICU stay) by PHASE-inferred haplotype in our study cohort. We found haplotypes 1 to 3 were associated with greater occurrence of prolonged ICU stay (Figure 2). Haplotypes 1 to 3 are uniquely tagged by the T allele of the 9545 T/G polymorphism, so we focused on the 9545 T/G genotype for further analyses. These data were best fit using a recessive model (TT genotype compared to TG plus GG genotypes), which was therefore used as the statistical analytic model throughout the study. The TT genotype occurred in 58% of patients. Genotypes of the 9545 T/G SNP were in Hardy Weinberg equilibrium. No significant associations between genotype and any clinical or intermediate phenotype were observed for any of the three other htSNPs (data not shown).

### Baseline and peri-operative characteristics

There were no significant differences between patients homozygous for the T allele of the IL-18 9545 T/G SNP versus patients having a GT or GG genotype in baseline characteristics (Table 1) except for body mass index (BMI) (28 kg/m^2 ^for patients with TT genotype, 28 kg/m^2 ^for patients with GT genotype, 26 kg/m^2 ^for patients with GG genotype, p = 0.022). There were no significant differences by genotype in peri-operative management, including use of aprotinin (TT 15%, GT+GG 10%, p = 0.10), amicar (TT 39%, GT+GG 38%, p = 0.99), or protamine (TT 0%, GT+GG 1%, p = 0.18) in our study cohort. Similarly, there were no differences by genotype in the use of vasodilators dobutamine (TT 71%, GT+GG 75%, p = 0.25) or milrinone (TT 11%, GT+GG 13%, p = 0.47) in the perioperative period. In addition there was no difference in vasopressor use (receiving norepinephrine at 4, 12 and 24 hours: TT 7.8%, 5.2%, 2.1%, GT+GG 7.2%, 5.4%, 1.1%, respectively).

**Table 1:** Baseline characteristics by IL-18 9545 genotype

	9545 TT	9545 GT	9545 GG	Total	p value
Number (%)	383 (58)	225 (34)	50 (8)	658	
Age (years)	65 ± 1	66 ± 1	66 ± 1	66 ± 1	0.655
Male sex (%)	77	77	70	76	0.544
Body mass index (kg/m^2^)	28 ± 1	28 ± 1	26 ± 1	28 ± 1	0.022
Ejection fraction (%)	49.2 ± 0.7	48.8 ± 0.9	51.3 ± 2.1	49.3 ± 0.5	0.529
Renal dysfunction (%) (Creatinine > 200 μmol/L)	3.6	2.2	0.0	2.9	0.266
Diabetes (Types I & II) (%)	29	28	27	28	0.969
Smoking (%)	32	41	36	32	0.129
Anti-hypertensive use (%)	65	61	58	62	0.495
Angiotensin-converting enzyme II inhibitor use (%)	48	54	50	52	0.447
Beta-blocker use (%)	58	60	56	59	0.745
Aspirin use (%)	59	59	60	59	0.992
Duration of surgery (hours)	4.4 ± 0.1	4.5 ± 0.1	4.4 ± 0.1	4.5 ± 0.1	0.728
Duration of bypass (hours)	1.8 ± 0.1	1.9 ± 0.1	1.8 ± 0.1	1.8 ± 0.1	0.544
Cross clamp time (hours)	1.3 ± 0.1	1.4 ± 0.1	1.4 ± 0.1	1.4 ± 0.1	0.143

Continuous variables are reported as mean ± standard error of the mean.

### Primary clinical phenotype

**Figure 3:**
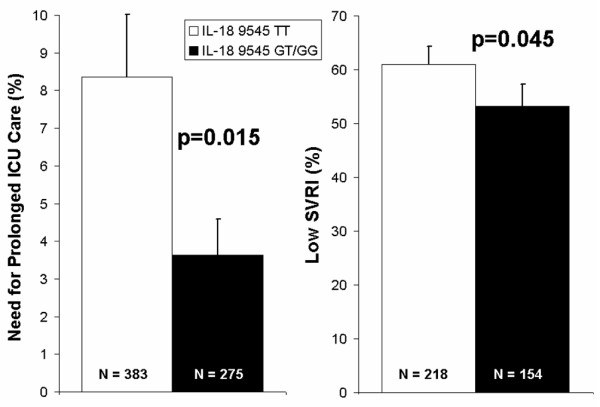
**Prolonged intensive care unit (ICU) stay and low Systemic Vascular Resistance Index (SVRI) after cardiopulmonary bypass (CPB) by interleukin (IL)-18 9545 T/G genotype in Caucasian patients who underwent on-pump CPB surgery**. Percentage of patients having prolonged ICU stay (greater than 72 hours) are indicated on the first Y-axis and percentage of patients having low SVRI after CPB surgery are indicated on the second Y-axis. IL-18 9545 T/G genotypes are indicated on the X-axis. Patients homozygous for the T allele of the IL-18 9545 T/G SNP had significantly greater occurrence of prolonged ICU stay and significantly increased occurrence of low SVRI after CPB compared with all others (GT and GG genotypes).

In our cohort of Caucasian patients who had CPB surgery, patients homozygous for the T allele of the IL-18 9545 T/G SNP had increased occurrence of prolonged ICU stay (greater than 72 hours) after CPB surgery: 32 of 383 (8.4%) for TT versus 10 of 275 (3.6%) for TG or GG (p = 0.015; Figure 3). The overall occurrence of prolonged ICU stay was 42 out of 658 patients (6.4%). These differences remained significant (p = 0.012) using logistic regression adjusted for age, gender, duration of bypass and baseline organ function (ejection fraction, diabetes, renal dysfunction; Table 2). The sensitivity of the TT genotype to predict prolonged ICU stay is (or true positive rate) 76% and the specificity (or true negative rate) is 43%.

**Table 2:** Logistic regression parameters. The outcome of logistic regression is shown for a model where intensive care unit (ICU) stay more than three days is the dependent variable (primary outcome). The covariates entered into the model were IL-18 9545 genotype, age, gender, duration of bypass, pre-operative ejection fraction and whether the patient had diabetes or renal dysfunction. Renal dysfunction was defined as baseline creatinine more than 200 μmol/L

Covariate	Relative risk of ICU stay > three days	95% confidence interval	p value
IL-18 9545 TT versus TG/GG genotype	3.01	1.28 to 7.10	0.012
Age (per year)	1.01	0.98 to 1.04	0.608
Gender (female)	0.95	0.40 to 2.27	0.905
Duration of bypass (per hour)	2.09	1.49 to 2.93	0.000
Ejection fraction (per %)	0.97	0.95 to 1.0	0.026
Diabetes	1.10	0.50 to 2.43	0.806
Renal dysfunction	1.77	0.36 to 8.63	0.482

### Secondary clinical phenotype

In these Caucasian patients undergoing elective on-pump coronary bypass graft surgery (CPB), those patients homozygous for the T allele of the IL-18 9545 T/G SNP had increased frequency of two consecutive SVRI measurements less than 1800 dyne.s/cm^5^/m^2^. The TT genotype was associated with the increased frequency of two SVRI measurements less than 1800 (62%) compared with the GT+GG genotypes (53%) (Figure 3). These differences remained significant (p = 0.045) using logistic regression adjusted for age, gender and duration of bypass (Table 3). The difference in SVRI was not accounted for by differences in post-operative use of vasodilating inotropes or the vasopressor norepinephrine (above). The fraction of patients having two or more Systemic Inflammatory Response Syndrome (SIRS) criteria at 24 hours was 24% in TT patients and 20.7% in GT+GG patients. The heart rate component of SIRS scoring was significantly different with TT patients having a heart rate at 24 hours of 78.4 ± 0.7 compared with 75.8 ± 0.7 for GT+GG patients (p = 0.016). A trend to increased numbers of TT patients remaining on the ventilator at 24 hours (TT 10.2%, GT+GG 8.3%), increased numbers of patients having a rise in creatinine of more than 50 μmol/L at 24 hours (TT 4.1%, GT+GG 2.5%) and increased mortality (TT 1.8%, GT+GG 1.1%) was observed, although the numbers of patients falling into these categories was too low for these differences to be statistically significant.

**Table 3:** Logistic regression parameters. The outcome of logistic regression is shown for a model where low post-cardiopulmonary bypass (CPB) Systemic Vascular Resistance Index (SVRI) is the dependent variable (secondary outcome). The covariates entered into the model were IL-18 9545 genotype, age, gender and duration of bypass.

Covariate	Relative risk of low post-CPB SVRI	95% confidence interval	p value
IL-18 9545 (TT versus TG/GG genotype)	1.57	1.01 to 2.44	0.045
Age (per year)	1.01	0.98 to 1.03	0.480
Gender (female)	0.70	0.39 to 1.27	0.703
Duration of bypass (per hour)	1.03	0.66 to 1.62	0.889

### Intermediate phenotype

**Figure 4:**
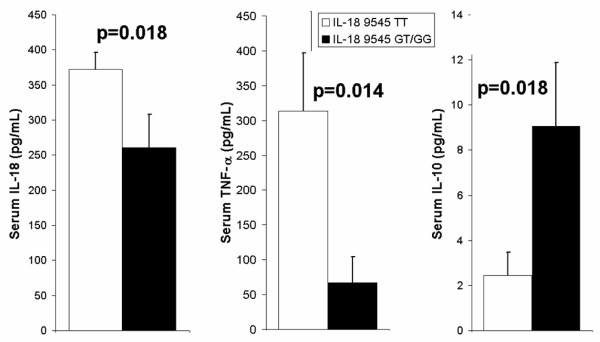
**Serum concentrations of interleukin (IL)-18, tumour necrosis factor (TNF)-α and IL-10 by IL-18 9545 T/G genotype in Caucasian patients who underwent on-pump cardiopulmonary bypass (CPB) surgery**. Serum concentrations of cytokines (pg/mL) are indicated on the y-axes, with IL-18 9545 T/G genotype indicated in the legend. Serum cytokine concentrations were measured 24 hours post-CPB. Patients homozygous for the T allele of the IL-18 9545 T/G SNP had significantly higher serum IL-18 and TNF-α concentrations and significantly lower serum IL-10 concentrations 24 hours post-CPB.

In a subgroup of patients for whom serum cytokine measurements were available, a significant difference was found in the concentration of IL-18 24 hours post-CPB surgery by genotype of the IL-18 9545 T/G polymorphism. Patients homozygous for the T allele had a significantly greater mean serum IL-18 concentration (372 ± 24 pg/mL) compared with those with the GT or GG genotypes (260 ± 48 pg/mL, p = 0.018; Figure 4).

A significant difference in serum concentration of TNF-α by IL-18 9545 T/G genotype was observed 24 hours post-operatively. Patients homozygous for the T allele of the IL-18 9545 T/G SNP had a significantly higher serum concentration of TNF-α at this time point (314 ± 83 pg/mL) compared with all other patients (67 ± 37 pg/mL, p = 0.014; Figure 4).

A significant difference in serum IL-10 levels was observed at 24 hours post-CPB by IL-18 9545 T/G genotype. Patients homozygous for the T allele had significantly lower serum IL-10 levels 24 hours post-CPB (2.4 ± 1.0 pg/mL) compared with all other patients (9.1 ± 2.8 pg/mL, p = 0.018; Figure 4).

## Discussion

We have identified an association between the TT genotype of the novel 9545 T/G polymorphism of the IL-18 gene and adverse outcome after CPB surgery. The TT genotype was found to be associated with increased occurrence of prolonged ICU stay and of two consecutive SVRI measurements less than 1800 dyne.s/cm^5^/m^2^. A biologically plausible explanation for these findings is that the TT genotype of the 9545 T/G htSNP was also associated with higher serum concentration of IL-18, higher serum TNF-α concentration and lower serum concentration of IL-10 in our cardiac surgical population. Prolonged ICU stay is reported to be correlated with risk factors for poor outcome after CPB surgery [11]. Low systemic vascular resistance, defined as two consecutive SVRI measurements less that 1800 dyne.s/cm^5^/m^2^, is a marker of poor vascular tone after CPB, related to the systemic inflammatory response [13]. Greater serum concentrations of IL-18 [2] and TNF-α [1] and lower serum levels of IL-10 [1] have been associated with increased prevalence of complications after CPB, and may be indicative of a prominent pro-inflammatory state.

Production of humoral inflammatory mediators and priming of neutrophils by exposure to the CPB apparatus enables a 'post-pump' syndrome characterised by a systemic inflammatory response syndrome and its anti-inflammatory counterpart, termed the compensatory anti-inflammatory response syndrome [20]. IL-18 plays a central role in regulating and balancing these responses. IL-18 regulates the expression of the potent pro- and anti-inflammatory mediators TNF-α [4] and IL-10 [5]. In accord with this, we found that the TT genotype was associated with an increased serum IL-18 concentration and also with increased serum TNF-α and decreased serum IL-10. The increased serum TNF-α and decreased serum IL-10 levels are associated with increased organ dysfunction [10]. Therefore, our results are consistent with the hypothesis that the IL-18 9545 TT genotype leads to increased production of serum IL-18, subsequently leading to increased serum TNF-α and decreased serum IL-10, causing increased organ dysfunction and increased occurrence of prolonged ICU stay.

Polymorphisms in the IL-18 gene have been studied for association with inflammatory conditions such as type 1 diabetes [3] and sepsis [21]. The -607 C/A and -137 G/C promoter SNPs have been found to be associated with a susceptibility to type 1 diabetes such that the C allele of -137 was found to be a risk allele and the A allele of -607 was found to be a protective allele [3]. Polymorphisms in the IL-18 gene have also been associated with alterations in serum concentrations of IL-18 [22]. None of the previous literature reports an association between the 9545 T/G SNP and outcomes, or between this SNP and serum cytokine levels. The previous SNPs, -607 C/A and -137 G/C are not in significant linkage disequilibrium with the 9545 T/G SNP (Figure 1). Neither of these previously reported SNPs were in association with our primary or secondary clinical or intermediate phenotypes.

The 9545 T/G SNP of the IL-18 gene is located within intron 2 and therefore may not be the causal SNP with regard to clinical outcomes or intermediate phenotypes. Strong linkage disequilibrium exists between the 9545 T/G SNP and several other SNPs within the IL-18 gene (Figure 1): -2163 C/A (rs5744222) in the promoter; 790 G/T (n/a) and 8936 A/G (rs4988359) in intron 1; 11024 G/C (rs1834481), 12003 T/C (rs5744256) and 13084 G/C (rs5744258) in intron 3; and 17980 G/C (rs5744276) in intron 5. Promoter SNPs are likely to have an effect by disrupting or creating binding sites for transcription factors, thus altering levels of intracellular transcript and/or extracellular protein [23]. The -2163 C/A SNP, however, is not found to be located within a putative transcription factor binding site, nor does the rare allele create one, based on a scan of the promoter region of the IL-18 gene using TESS (transcriptional element search software). Intronic SNPs can affect splicing of the pre-mRNA, resulting in alternative splice variants of proteins; however, most intronic SNPs thought to have these effects are observed to lie within about 20 bp of intron/exon boundaries [24]. The 9545 T/G SNP itself does not lie so close to such a boundary, nor do any of the SNPs in LD with 9545 mentioned above. Linkage disequilibrium can carry over the putative gene boundary, and may allow the 9545 T/G SNP to be tightly correlated with a causal SNP up- or downstream of the IL-18 gene, one which may have a causal effect on clinical and intermediate phenotypes.

Haplotypes represent a powerful method of selecting SNPs for genotyping based on linkage disequilibrium. The IL-18 gene has 56 polymorphic loci in Caucasians according to sequencing data from the University of Arizona's Innate Immunity Program in Genomic Applications website. The SNPs at positions -607 and -137 relative to the start of translation are literature SNPs, and so make good choices for genotyping in an inflammatory-related population such as CPB patients. The positions 8148 and 9545 are not previously reported SNPs, however, and so would not have been chosen for genotyping without some method of selecting SNPs. By selecting htSNPs to maximise information while minimising the number of SNPs to be genotyped, we have queried the underlying haplotype structure while using relatively few polymorphic loci. Although this marker-style approach to disease association is useful, it does not address mechanism or functionality of the polymorphism itself.

Our study has several strengths. The use of haplotypes to choose htSNPs has the benefit of not being restricted to literature SNPs for disease association studies. The large sample size allows for statistical power to detect associations of modest effect and the limitation of sampling to Caucasians patients reduces the likelihood of type I error due to population admixture.

One of the main weaknesses of our study design is that we do not identify the causative SNP for worse clinical outcome after CPB. Linkage disequilibrium existing within and possibly beyond the IL-18 gene suggests that polymorphic loci in linkage disequilibrium with the T allele of the 9545 T/G SNP could contribute to detrimental effects after CPB surgery. We have limited our study to a single cohort; therefore this arising hypothesis should be tested in other cohorts to ensure reproducibility.

## Conclusions

In the present study the TT genotype of a novel polymorphism of the IL-18 gene, 9545 T/G, was associated with greater occurrence of prolonged ICU stay after CPB surgery, greater frequency of low SVRI (two consecutive SVRI measurements < 1800 dyne.s/cm^5^/m^2^), higher serum concentrations of cytokine IL-18, higher serum concentrations of the cytokine TNF-α and lower serum concentrations of the anti-inflammatory cytokine IL-10. These widely varied markers of intensity of recovery post-CPB indicate this genotype is potentially a risk factor for patients undergoing CPB surgery.

## Key messages

• The TT genotype of the IL-18 9545 T/G polymorphism is associated with an increased occurrence of prolonged stay in the ICU post-surgery.

• The same genotype was associated with increased IL-18 levels.

• The increase in IL-18 levels associated with the TT genotype appeared to result in increased pro-inflammatory TNF-α levels and decreased anti-inflammatory IL-10 levels; TNF-α and IL-10 having previously been shown to be regulated by IL-18 in this way.

• The pro-inflammatory balance (as indicated by increased TNF-α and decreased IL-10) may account for the adverse clinical outcomes associated with the TT genotype post surgery.

## Abbreviations

BMI: body mass index; bp: base pair; CI: cardiac index; CPB: cardiopulmonary bypass; CVP: central venous pressure; ELISA: enzyme-linked immunosorbent assay; htSNP: haplotype tag single nucleotide polymorphism; ICU: intensive care unit; IL: interleukin; INF-γ: interferon-gamma; MAP: mean arterial pressure; PCR: polymerase chain reaction; SE: standard error; SIRS: Systemic Inflammatory Response Syndrome; SNP: single nucleotide polymorphism; SVRI: Systemic Vascular Resistance Index; TNF-α: Tumour necrosis factor-alpha

## Competing interests

The authors declare that they have no competing interests.

## Authors' contributions

DS contributed to experimental design, data collection, genotyping and protein measurement, conducted the primary analysis of data and wrote the initial draft of the manuscript. AS assisted in data collection, in genotyping and in protein measurement. JR and SL contributed to experimental design and data collection. KRW contributed to experimental design, data collection and data analysis. All authors read, approved and contributed to the final draft of the manuscript.
